# Enabling Policy Planning and Innovation Management through Patent Information and Co-Authorship Network Analyses: A Study of Tuberculosis in Brazil

**DOI:** 10.1371/journal.pone.0045569

**Published:** 2012-10-03

**Authors:** Alexandre Guimarães Vasconcellos, Carlos Medicis Morel

**Affiliations:** 1 National Institute of Industrial Property (INPI), Rio de Janeiro, Brazil; 2 National Institute for Science and Technology on Innovation on Neglected Diseases (INCT/IDN), Center for Technological Development in Health (CDTS), Oswaldo Cruz Foundation (Fiocruz), Rio de Janeiro, RJ, Brazil; Beijing Institute of Microbiology and Epidemiology, China

## Abstract

**Introduction:**

New tools and approaches are necessary to facilitate public policy planning and foster the management of innovation in countries' public health systems. To this end, an understanding of the integrated way in which the various actors who produce scientific knowledge and inventions in technological areas of interest operate, where they are located and how they relate to one another is of great relevance. Tuberculosis has been chosen as a model for the present study as it is a current challenge for Brazilian research and innovation.

**Methodology:**

Publications about tuberculosis written by Brazilian authors were accessed from international databases, analyzed, processed with text searching tools and networks of coauthors were constructed and visualized. Patent applications about tuberculosis in Brazil were retrieved from the Brazilian National Institute of Industrial Property (INPI) and the European Patent Office databases, through the use of International Patent Classification and keywords and then categorized and analyzed.

**Results/Conclusions:**

Brazilian authorship of articles about tuberculosis jumped from 1% in 1995 to 5% in 2010. Article production and patent filings of national origin have been concentrated in public universities and research institutions while the participation of private industry in the filing of Brazilian patents has remained limited. The goals of national patenting efforts have still not been reached, as up to the present none of the applications filed have been granted a patent. The analysis of all this data about TB publishing and patents clearly demonstrates the importance of maintaining the continuity of Brazil's production development policies as well as government support for infrastructure projects to be employed in transforming the potential of research. This policy, which already exists for the promotion of new products and processes that, in addition to bringing diverse economic benefits to the country, will also contribute to effective dealing with public health problems affecting Brazil and the World.

## Introduction

The process of developing new pharmaceuticals is expensive and time consuming. The data extant concerning this process are the result of innumerable studies suggesting that it can range from USD 300 million to USD 1 billion, and take up to 12 years to bring a new medication to market [1]–[4].

To attend to the public health needs of their populaces and spur the process of innovation in the health area, decision makers in both the public and private spheres must analyze innumerable factors to manage the reduction of uncertainties and the optimization of results.

These activities are of particular importance in planning the combat of the so-called neglected diseases which are exclusive to, or prevalent in, developing countries and poverty-stricken areas. These afflictions are not a priority for the pharmaceutical and biotechnology industries which are responsible for the development of medications, vaccines, and diagnostic kits [5] as they do not represent a significant revenue stream.

In this context, a country like Brazil, which possesses significant scientific development in this area, and has some industries and institutions capable of carrying out research and development (R&D), and both great biodiversity and consumer market potential, can develop the capacity to participate directly in the search for new bioactive molecules and their transformation into medications and diagnostic kits for existing diseases. All things being equal, they should optimize the allocation of their limited resources, as well as establishing public policies that articulate and coordinate the diverse actors who make up the network [6], [7].

### Why tuberculosis?

Tuberculosis is a global affliction, and the WHO estimates that it affects two billion people, which means that a third of the world's population is infected with the bacillus *Mycobacterium tuberculosis* (M.tb). The bacillus is capable of lying dormant in a human body for years until an individual's immunity system becomes unable to ward off the infection and incapable of destroying the bacillus. While the majority of people with the latent infection never develop the active form of the disease, between 5% and 10% of the carriers will be afflicted by it during their lives [8]. In 2010 alone it is estimated that globally over 1.45 million people died as a direct result of tuberculosis while another 8.8 million developed the active form of the disease. Although in some parts of the world incidence rates are falling, in those where poverty is prevalent and HIV/AIDS rates are high the disease has maintained a continuous growth rate, and it is the principal infectious disease responsible for AIDS/HIV mortalities. This global pandemic disease is deepening the chasm which separates rich and poor countries and further accentuating global social stress. For example, in 2009 alone, almost 10 million children were orphaned by parental mortalities caused by tuberculosis [9].

In the case of Brazil, it can be observed that the tuberculosis incidence rate has been in decline. There were 51.4 cases per 100,000 inhabitants in 1990 but by 2007, the rate had fallen to 38.4 per 100,000. Investments in tuberculosis (including medication) have also increased substantially, jumping from USD 9.3 million in 2000 to USD 69.1 million in 2008 (National Tuberculosis Control Program, 2010). Despite these advances, currently there are around 5,000 reported deaths due to tuberculosis each year [9].

The therapeutic arsenal in current use for the treatment of tuberculosis, the so-callled first-line drugs, consists of four: isoniazid, ethambutol, pirazimide, and rifampicin which were developed in 1952, 1962, 1954, and 1963 respectively. A treatment cycle with these drugs takes from six to nine months, but those patients who are unable to complete the entire treatment cycle, currently around 8% in Brazil [10], may acquire resistant strains necessitating an additional two years of treatment with the so-called second-line drugs, which usually have serious side-effects. It is important to emphasize that worldwide in 2010 there were an estimated 650,000 cases of multiple drug-resistant tuberculosis (MDR-TB) and that M.tb has recently developed new forms of resistances to known drugs named extensively drug-resistant tuberculosis (XDR-TB) and totally drug-resistant tuberculosis (TDR) [11]. Thus, it has been confirmed that the tuberculosis drugs available are inadequate to meet the various inherent treatment challenges which is what makes the development of new biomedical technologies, new drugs and new drug regimens such a vital priority in global control and eradication programs [12], [13].

In line with the objectives of the United Nations, which has set a goal of drastically reducing the tuberculosis incidence rate by 2015, several initiatives and partnerships were created to deal with this challenge, such as: TB Alliance, launched in 2000; FIND, Foundation for Innovative New Diagnostics, launched in 2003; Stop TB initiative, created by the World Health Organization in 2006. They aim to provide universal access to high quality diagnostic tools and patient-centered treatment that can reduce the human suffering and socio-economic oppression associated with tuberculosis; protect the poor and populations vulnerable to TB, TB/HIV, and drug-resistant TB; and support the development of better tools, including new medicines and the facilitation of their rapid and efficient deployment.

There have already been initiatives to establish coordinated actions for the development of innovative technologies aimed at the combat of tuberculosis in Brazil. In the 1990s the “Fine Chemical Program to Combat Tuberculosis” (QTROP-TB) was carried out over a ten-year period through a partnership between the Financier of Studies and Projects (FINEP) and the Federal University of Rio de Janeiroro (UFRJ) with the collaboration of local industries as one of it main goals. Various activities, conducted with the aim of establishing a strategic vision for the future, mobilized more than 400 specialists and dozens of institutions from different economic sectors to better understand future technologies for combating tuberculosis [14]. However, the challenge to develop new low-cost and safe drugs, treatments, and rapid diagnostic kits still persists.

From a more general point of view, it should be emphasized that according to the Oslo Manual [15], there are two basic families of indicators for science and technology, which are directly relevant to innovation measurement: resources devoted to R&D and patent statistics. In addition, bibliometric and various other sorts of indicators offer complementary information, although much of this type of information is generally not easily available in an entrepreneurial environment.

To leverage structural projects that articulate competencies already installed in Brazil and identify bottlenecks in the path leading from the laboratory bench to market it is fundamental to identify some items. The most important ones are: (1) who are the authors publishing in the area?; (2) in which institutions are they located?; (3) which institutions seek patent protection for their inventions?; (4) does a correlation exist between the Brazilian institutions that publish and the ones that apply for patents?; and (5) what are the results of these patent applications? In addition to these items, it is important to study who is interested in this market in Brazil and, starting with this, identify some criteria such as possible partners, suppliers, and market niches to enter, among others. It is of great relevance to also verify: (6) who are the foreign applicants? and; (7) what countries are they from?

The objective of the present study is to respond to these questions by using two search tools that deal with data analyses in a consolidated manner: (i) the study of tuberculosis co-authorship networks and (ii) a survey of information found in patent applications about the theme in Brazil.

## Results and Discussion

### Tuberculosis co-authorship networks

**Figure 1:**
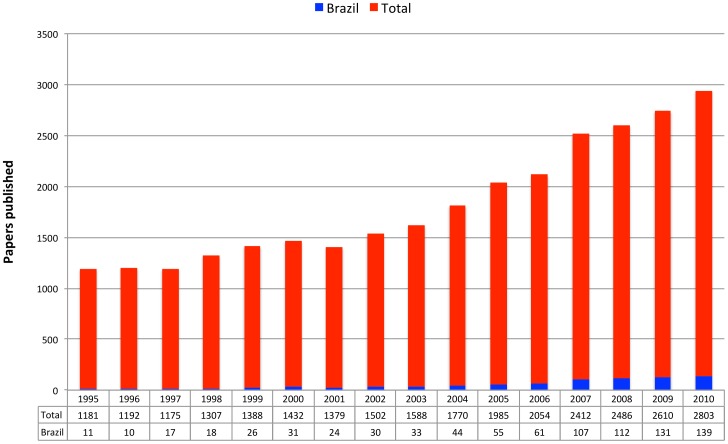
Papers published on tuberculosis during the period 1995–2010. Papers by Brazilian scientists are represented by the blue bars.

During the period from 1995 to 2010 there were 28,264 articles surveyed of which 860 had a Brazilian author. During this period the Brazilian production of scientific articles about tuberculosis increased more than twelve fold, the proportion of articles about TB by Brazilian researchers in relation to global production raising from 0.93% to 4.96% (Figure 1). Table 1 lists the journals that published five or more papers authored by at least one Brazilian researcher and their respective impact factor. Table 2 lists the most productive 25 researchers authoring these papers.

**Table 1:** Journals publishing 5 or more articles on tuberculosis with Brazilian authors, 1995–2010.

Journal	Impact factor	# Articles
Int. J. Tuberc. Lung Dis	2.731	79
Rev. Saude Publica	1.328	49
Mem. Inst. Oswaldo Cruz	2.147	44
J. Bras. Pneumol	1.391	27
J. Clin. Microbiol	4.153	27
Braz. J. Infect. Dis	1.005	23
Am. J. Respir. Crit. Care Med	11.080	17
Rev. Soc. Bras. Med. Trop	0.681	17
Chest	5.250	15
Brazilian J. Med. Biol. Res	1.129	14
Infect. Immun	4.165	14
Rev. Latino-Am. Enfermagem	0.625	14
Cad. Saude Publica	0.889	12
Braz. J. Microbiol	0.896	11
Int. J. Infect. Dis	1.938	11
Rev. Esc. Enferm. USP	0.375	11
Microbes Infect	3.101	9
Trans. Roy. Soc. Trop. Med. Hyg	2.162	9
Antimicrob. Agents Chemother	4.841	8
Clin. Exp. Immunol	3.360	8
Clin. Immunol	3.893	8
J. Infect. Dis	6.410	8
Am. J. Trop. Med. Hyg	2.592	7
Arq. Neuro-Psiquiatr	0.722	7
PLoS One	4.092	7
Protein Expr. Purif	1.587	7
Tuberculosis	3.474	7
Vaccine	3.766	7
Biochemistry	3.422	6
Bioorg. Med. Chem	2.921	6
J. Immunol	5.788	6
Lancet	38.278	6
Arch. Biochem. Biophys	2.935	5
BMC Infect. Dis	3.118	5
Clin. Infect. Dis	9.154	5
Immunology	3.321	5
J. Microbiol. Methods	2.086	5
J. Venom. Anim. Toxins Trop. Dis	0.429	5
Scand. J. Immunol	2.230	5

The impact factor weighted average (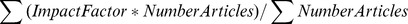) is 3.141.

**Table 2:** Top 25 researchers publishing articles on tuberculosis with Brazilian authors, 1995–2010.

Rank	Researcher	Institution	# Articles
1	Kritski, Afranio Lineu	UFRJ	82
2	Basso, Luiz Augusto	PUCRS	49
3	Santos, Diogenes Santiago	PUCRS	47
4	Silva, Celio Lopes	USP	47
5	Dietze, Reynaldo	UFES	33
6	Netto, Antonio Ruffino	USP	33
7	Conde, Marcus Barreto	UFRJ	27
8	Palaci, Moises	UFES	27
9	Scatena Villa, Tereza Cristina	USP	23
10	Chaisson, Richard E	Johns Hopkins Univ	22
11	Feres Saad, Maria Helena	Fiocruz	21
12	Fonseca, Leila S	UFRJ	20
13	Mello, Fernanda C Queiroz	UFRJ	19
14	Faccioli, Lucia Helena	USP	18
15	Maciel, Ethel Leonor Noia	UFES	18
16	Palma, Mario Sergio	UNESP	18
17	Suffys, Philip Noel	Fiocruz	18
18	Trajman, Anete	UGF	18
19	Deperon Bonato, Vania Luiza	USP	17
20	Johnson, John L	Case Western Reserve Univ	17
21	Lapa e Silva, Jose Roberto	UFRJ	17
22	Ho, John L	Cornell Univ	16
23	Rosa Rossetti, Maria Lucia	FEPPS	15
24	Ferrazoli, Lucilaine	IAL	14
25	Ribeiro, Marta Osorio	FEPPS	14

Abbreviations of Brazilian institutions: FEPPS: Fundação Estadual de Produçãão e Pesquisa em Saúde (State Foundation for Production and Health Research); Fiocruz: Oswaldo Cruz Foundation; PUCRS: Pontifical Catholic University of Rio Grande do Sul; UFES: Federal University of Espírito Santo; UFRJ: Federal University of Rio de Janeiro; UGF: Gama Filho University; UNESP: Universidade Estadual Paulista; USP: University of São Paulo;

The 25 institutions most featured, in terms of tuberculosis article publication involving Brazilian authors, are listed in Table 3. It should be noted that the ten Brazilian institutions that published the most articles about TB are located in the southern and southeastern regions of the country, a consequence of the great asymmetry of economic development, scientific production and knowledge of the disease among the five geographic regions of Brazil (North, Northeast, Midwest, Southeast and South; http://www.brasil.gov.br/sobre/geography/politic-geography/regions-of-brazil-1/br_model1?set_language=en).

**Table 3:** Top 25 institutions publishing articles on tuberculosis with Brazilian authors, 1995–2010 (Foreign institutions in italics).

Rank	Institution	# Articles
1	University of São Paulo (USP)	207
2	Federal University of Rio de Janeiro (UFRJ)	187
3	Oswaldo Cruz Foundation (Fiocruz)	169
4	Federal University of Rio Grande do Sul (UFRGS)	65
5	Catholic University of Rio Grande do Sul (PUCRS)	51
6	São Paulo State University (UNESP)	51
7	Instituto Adolfo Lutz	43
8	Federal University of Espírito Santo (UFES)	43
9	*John Hopkins University*	33
10	Federal University of Minas Gerais (UFMG)	32
11	Campinas State University (UNICAMP)	31
12	Federal University of Bahia (UFBA)	30
13	Federal University of Pernambuco (UFPE)	23
14	*Cornell University*	22
15	Fluminense Federal University (UFF)	21
16	*Case Western Researve University*	20
17	*London School of Hygiene and Tropical Medicine*	19
18	Gama Filho University (UGF)	19
19	Federal University of São Paulo (UNIFESP)	17
20	*University of California, Berkeley*	16
21	Federal University of Rio Grande (FURG)	15
22	Federal University of Ceará (UFCE)	15
23	*University of Arkansas*	15
24	*Institute of Tropical Medicine, Antwerp*	14
25	State Foundation of Production and Health Research	13

Scientific co-authorship networks are a powerful instrument for analyzing scientific and technological collaborations and partnerships [16]–[18], and they complement the classic indicators employed in bibliometric studies such as the number of articles published during a specific period, impact factor, and index H, among others. Recently, Morel and collaborators applied this coverage to the planning, implementation, monitoring, and evaluation of neglected disease R&D programs [19].

**Figure 2:**
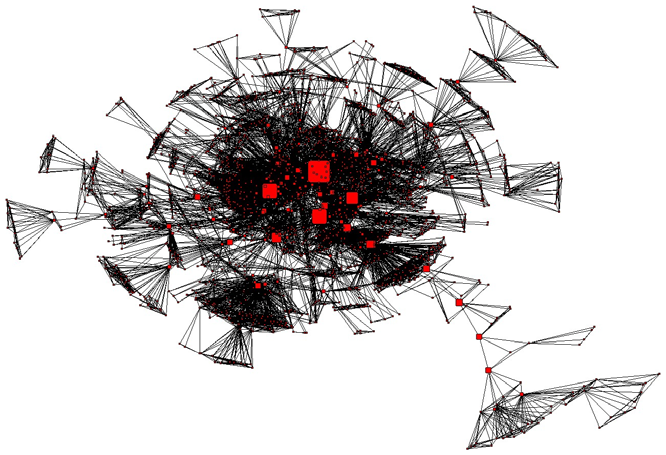
Major component of the co-authorship network of Brazilia scientists publishing on tuberculosis, 1995–2010. Node size proportional to the calculated betweenness centrality measure of authors (Table 3).

**Figure 3:**
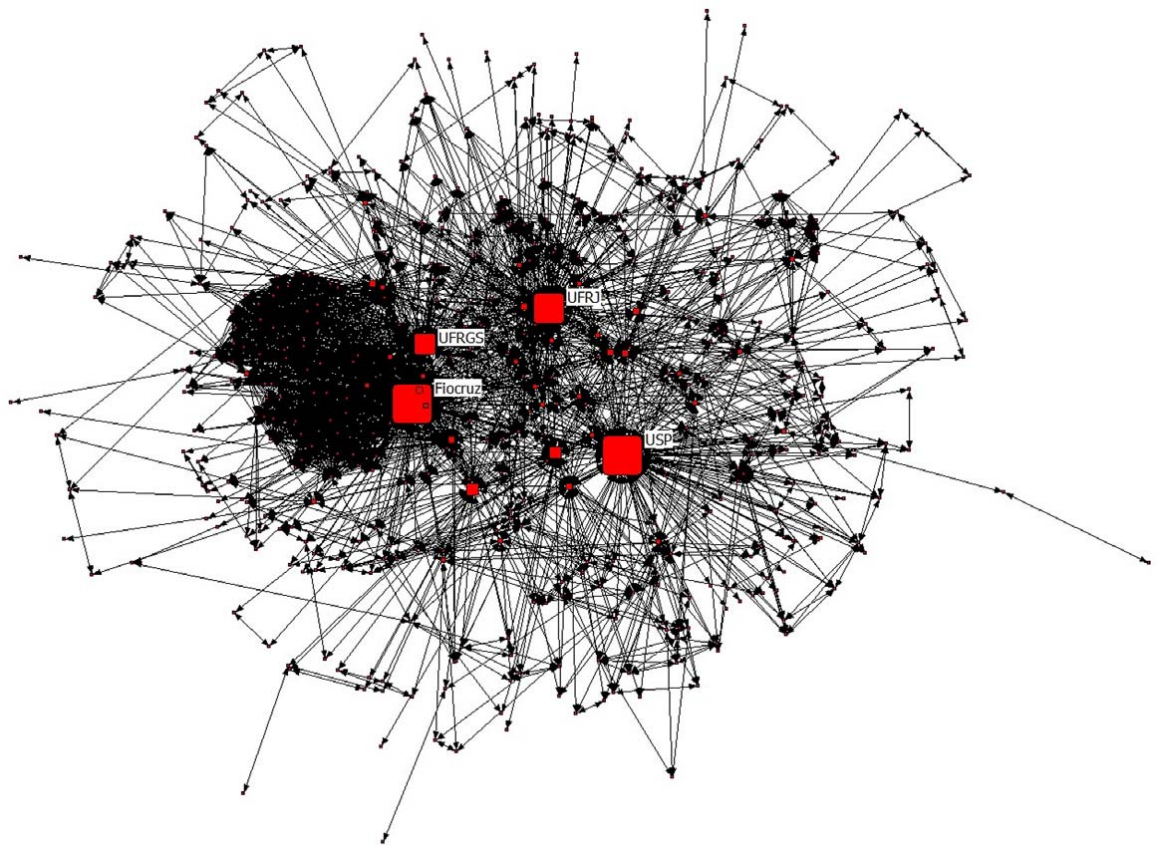
Major component of the network of Brazilian institutions working on tuberculosis, 1995–2010. Node size proportional to the calculated betweenness centrality measure of institutions. Four institutions have a central role in the network according to this indicator: University of São Paulo (USP), Oswaldo Cruz Foundation (Fiocruz), Federal University of Rio de Janeiro (UFRJ) and Federal University of Rio Grande do Sul (UFRGS).

The major components of the TB co-authorship networks of Brazilian scientists and their institutions are shown in Figures 2 and 3, respectively, where network node sizes are displayed in proportion to their “betweenness” (Tables 4 and 5), an indicator of the gatekeeper/broker role of the node in the network [20], [21]. The analysis of these networks shows that: (i) 14 authors are present in both Table 2 (authors ranked by total number of publications) and Table 4 (authors ranked by betweenness in the network); (ii) The four institutions that are the most productive in tuberculosis research in Brazil (Table 3) also represent the four most prominent gatekeeper nodes of the TB institutional network (Table 5).

**Table 4:** Rank of researchers publishing articles on tuberculosis with Brazilian authors, 1995–2010, according to the “betweeness” centrality measure.

Rank	Researcher	Betweenness
1	Kritski, Afranio Lineu	424364
2	Palaci, Moises	281152
3	Suffys, Philip	273810
4	Netto, Antonio Rufino	215079
5	Leao, Sylvia Cardoso	177922
6	Basso, Luiz Augusto	131645
7	Dietze, Reynaldo	130094
8	Santos, Diogenes Santiago	121802
9	Sant'Anna, Clemax Couto	108843
10	Caceres, RafaelAndrade	106431
11	Vargas, P.A.	99306
12	da Costa, P. Albuquerque	92166
13	Saldiva, P. H. N.	88560
14	Albuquerque, M.F.P.M.	88396
15	Hijjar, Miguel Aiub	85941
16	Silva, Celio Lopes	79736
17	Capelozzi, Vara Luiza	75932
18	Chaisson, Richard E.	73861
19	Cuevas, Luis Eduardo	73779
20	Conde, Marcus Barreto	69841
21	Fonseca, Leila S.	67021
22	Rodrigues, Laura Cunha	62399
23	Ribeiro, Marta Osorio	60738
24	Ferrazoli, Luciaine	56111
25	Lapa e Silva, José Roberto	54310

**Table 5:** Rank of institutions publishing articles on tuberculosis with Brazilian authors, 1995–2010, according to the “betweeness” centrality measure.

Rank	Institution	Betweenness
1	USP	24322
2	Fiocruz	23907
3	UFRJ	17946
4	UFRGS	12649

**Figure 4:**
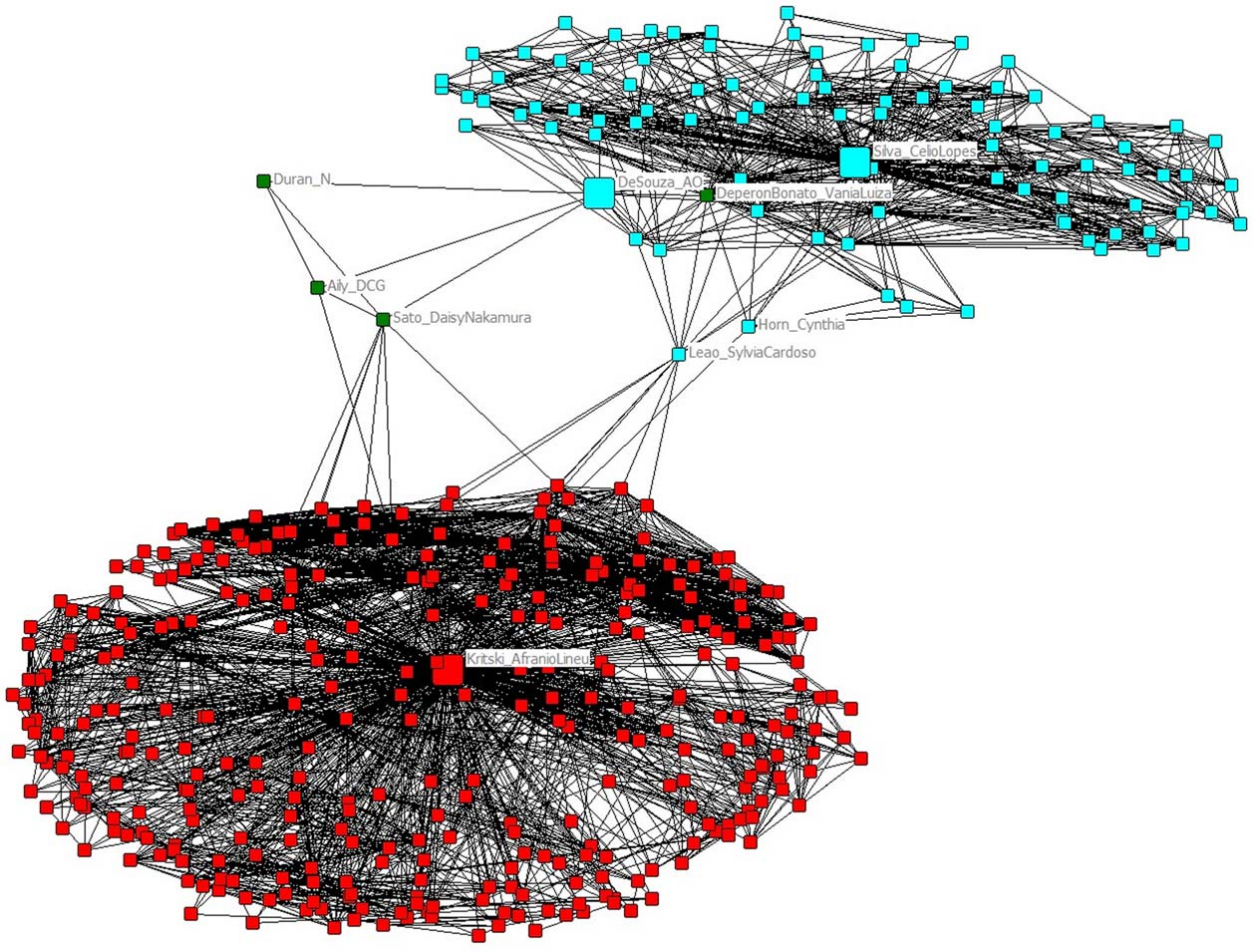
Co-authorship ego-network of the Brazilian scientists who published more papers (A. Kritsky) and filed more patent applications (C. Silva in collaboration with A. O. Souza) during the period 1995–2010.

Figure 4 displays the co-authorship ego-networks of the authors who have published most articles (A. Kritski) and filed most patent applications (C. Silva in collaboration with A. O. Souza, see below). Their respective networks are quite distinct, probably due to their different research subjects: TB treatment, diagnosis and control (AK) and vaccine R&D (CS/AOS). The only connections between these ego-networks are provided by few researchers (C. Horn, S. C. Leão, N. Duran, D. N. Sato, D. C. G. Aily), who work on subjects at the interface of these research fields. Further details about these scientists can be found at the “Plataforma Lattes” site of Brazil's National Council for Scientific and Technological Development, CNPq, a database with over two million curricula of scientists working in Brazil (http://lattes.cnpq.br/).

### Tuberculosis patent applications in Brazil

**Figure 5:**
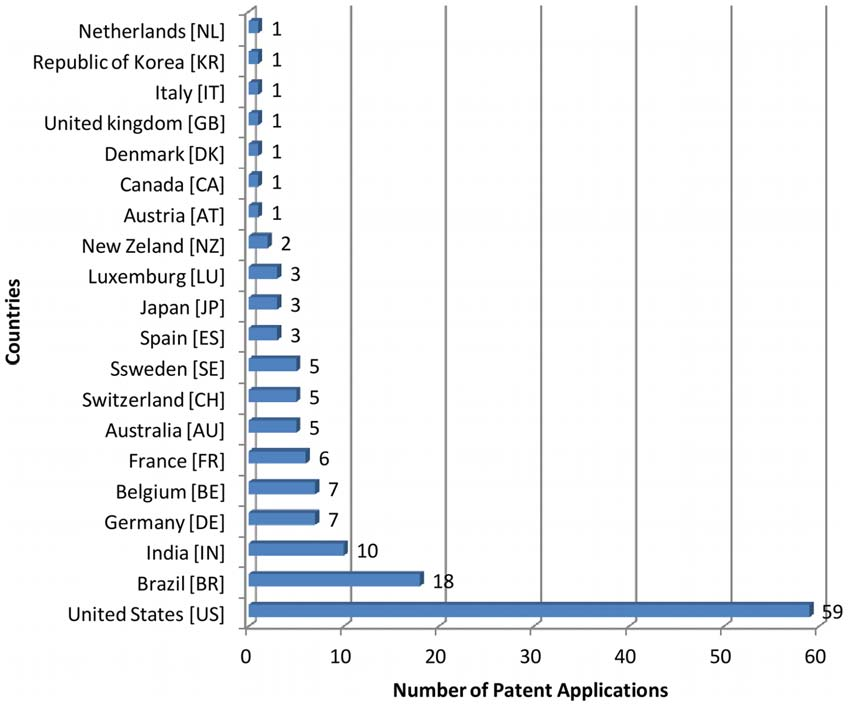
Nationalities of patent applicants of TB medicines or diagnostics applied in Brazil starting in 1995.

**Figure 6:**
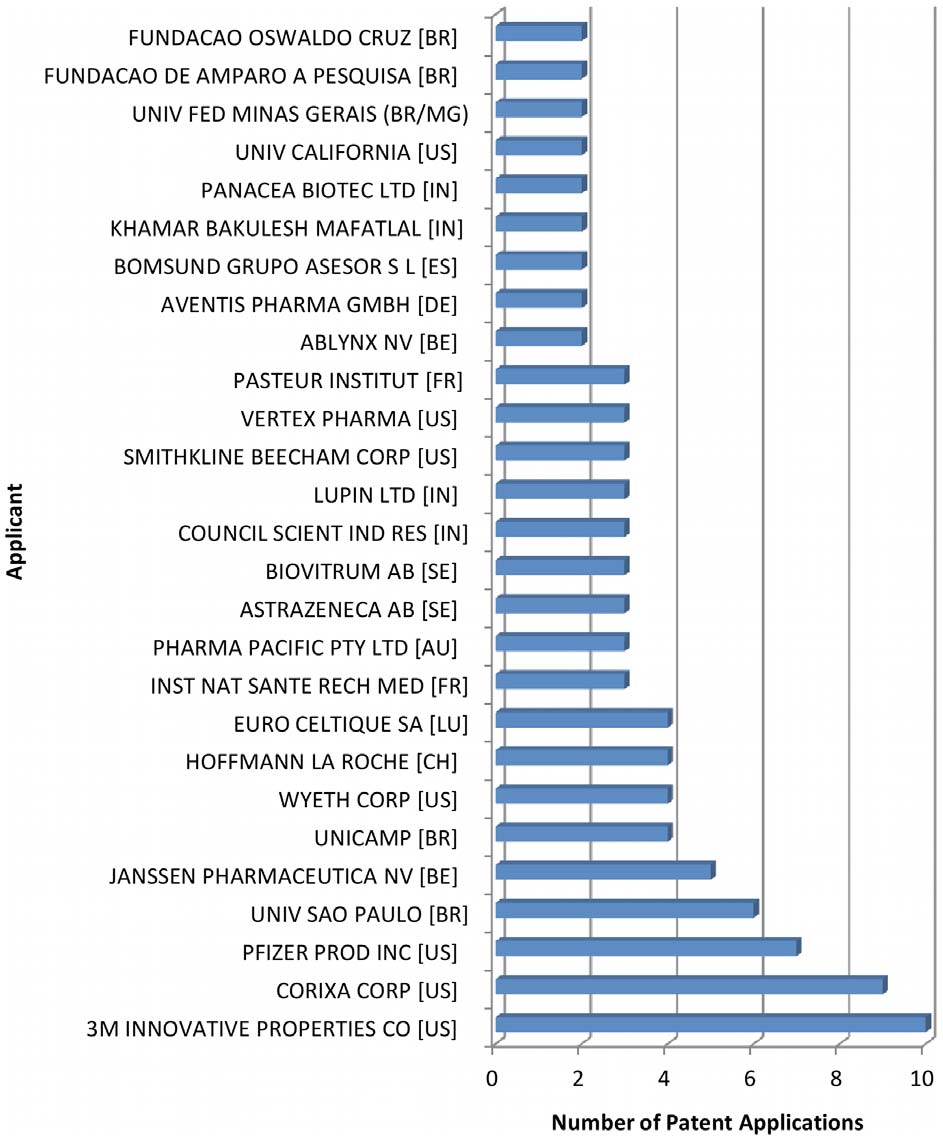
Applicants of patents related to TB medicines or diagnostics applied in Brazil starting in 1995. Only applicants of two or more patents are included in the figure.

Our search retrieved 138 patent applications filed in Brazil since 1995 related to tuberculosis therapeutics and/or diagnostics. The five countries most featured were the USA with 59 filings, Brazil with 18, India with 10, Germany with 7 and Belgium with 7 (Figure 5). Among the filers, the three main applicants were US companies (3 M Innovative Properties, Corixa Corporation (incorporated by GlaxoSmithKline in 2005), and Pfizer Products Inc.) (Figure 6).

The university that had the greatest number of applications was the University of São Paulo (USP) with six filings. All of them were co-titled with other universities, research institutions or a research support foundation, FAPESP (Research Support Foundation from the State of São Paulo) in this case. Two of the applications were originally exclusively from UNICAMP but later were shared with USP, and in one instance, a portion of one that was owned by a private individual was ceded to USP.

The two applications by research institutions pertain to the Oswaldo Cruz Foundation (Fiocruz), one of which is still being evaluated while the other one was denied.

**Figure 7:**
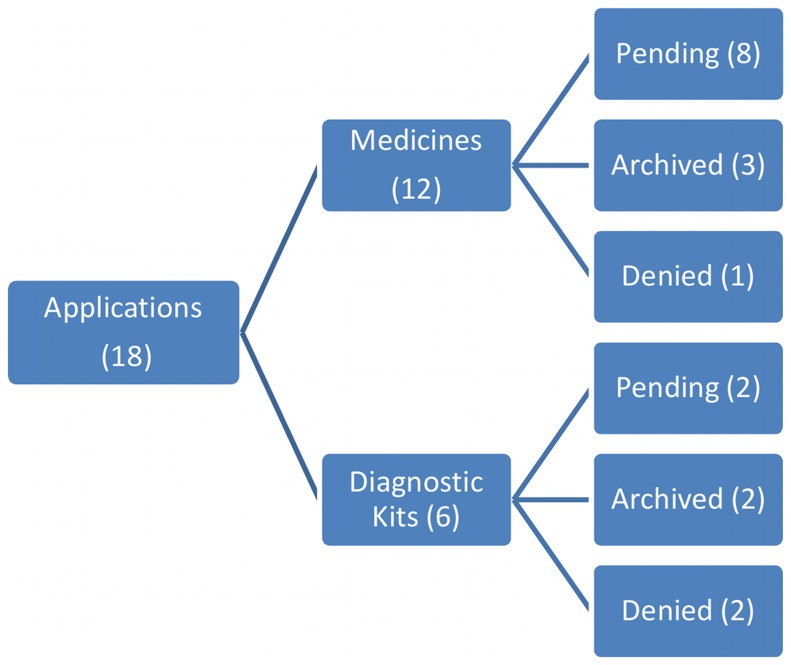
Status of patent applications related to TB medicines or diagnostics applied by Brazilian nationals, 1995–2008.

Of the 18 applications made by nationals, 12 referred to therapeutics and 6 to diagnostics. In the former group, 8 were being evaluated, 3 had been archived, and 1 had been denied, while in the latter group 2 were being evaluated, 2 had been archived, and 2 had been denied (Figure 7).The only application by a private individual was archived. Thus, it can be seen that to date there still has been no application of national origin granted.

Ten of the 18 patent applications are still under analysis. It is therefore important to note that a patent is a temporarily ceded property right, considered a movable property for all legal effects, and one that gives its owner the right to exclude third parties from the production, use, sales or importation of the protected product or process. A patent application is subjected to an exam of merit and to be granted it must fulfill the requirements of patentability. These include novelty, inventive step, and industrial application, in addition to being clearly and sufficiently described in a way which permits its reproduction by a specialized technician (Brazilian Law 9.279/96). The exclusive right to a patent only comes into force after the expedition of the patent certificate, and it is retroactive from the date of application. Before the expedition of the patent certificate is carried out, there can be only the expectation of the right. However, this does not impede the negotiation of the patent application, and thus it should be borne in mind that the risks of acquisition are greater in this situation and that this has implications in the valorization of the asset.

It can be observed that USP, the institution that published the most research during the period studied (1995–2010), was also the Brazilian one that filed the greatest number of patents. Celio Lopes Silva and Ana Olivia de Souza, from USP, were also the inventors with the largest number of Brazilian TB patent applications filed in Brazil during the period considered. It has also been verified that all the universities (USP, UNICAMP, and UFMG) and the research institution (Fiocruz) which filed more than two patents, also figured among the 11 entities that most published articles in the TB area. Thus, it can be observed that Brazil features a strong institutional association between scientific research, expressed by the number of scientific articles, and patent activity, expressed by the number of patent applications.

**Figure 8:**
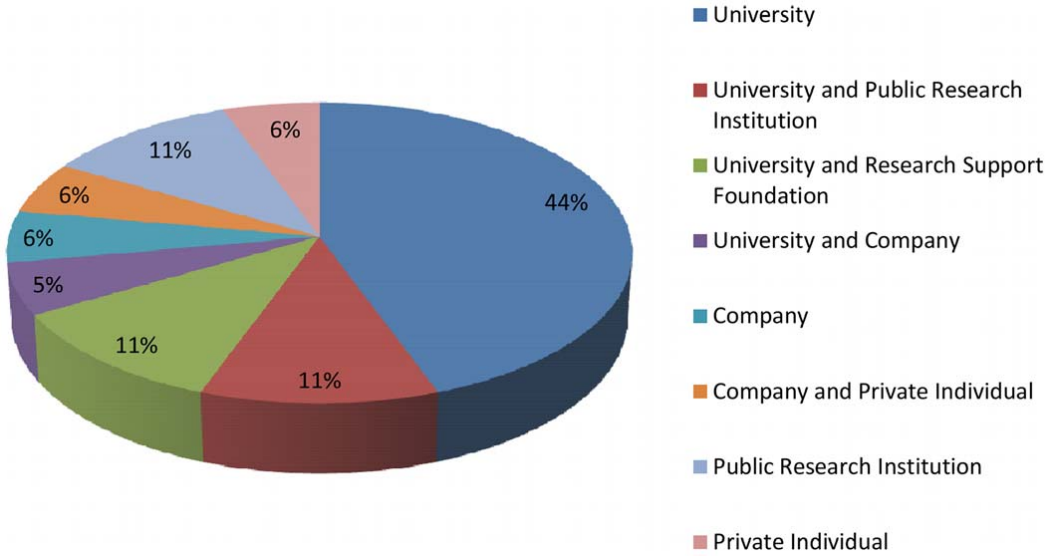
National applicants of patents in Brazil related to TB medicines or diagnostics starting in 1995.

Universities have played a very active role in innovative initiatives in the Brazilian TB field and are involved in 71% of all patent applications by nationals (Figure 8). However, it seems as though they are unable to overcome the barriers that exist to transform these results into products and bring them to the market. Weak participation by industry in patent applications in the area, only 17%, and poor cooperation between universities and companies (only one patent filing is co-titled 5%), demonstrates the lack of communication between academia and industry. This weak participation of industry enormously complicates the generation of innovation.

The combined analysis of publishing and patenting further reveals that Brazil has managed to consolidate its scientific research productivity in the TB area through universities and public research institutions over the past 15 years (Figures 1, 2, 3 and Tables 1, 2, 3, 4, and 5), and currently still relies on these very same actors to cope with the current challenges of technological innovation (Figures 6, 8).

In 2004, with the idea of fostering innovative public policies by creating a legal framework to solve these problems, Brazil passed the Law of Innovation (Law 10.973, December 2, 2004), which was designed to encourage strategic partnerships between universities, research institutions and private enterprises, and promotes the shared use of scientific and technological infrastructure by these three groups and thus fosters increased mobility of researchers inside the system [22]. Nevertheless, the creation of a legal framework favourable to innovation is incapable, in and of itself, of overcoming the systemic deficiencies occasioned by the incipient participation of private industry in the process of innovation in the area. It is also inappropriate to delegate to the universities the role of taking to market those inventions generated in an academic environment. To manage this aspect, policies have been created that focus on production development, and their main goals are an increase in fixed investments, a greater Brazilian participation in exportation, and expanding the private investments in R&D and making them more dynamic [23]. In this context, there are several ongoing structural projects and partnerships between the public and private sector - initiatives which have the objective of managing, and articulating research and technological development to produce medications and diagnostic kits aimed at serving the Brazilian public health sector, and, in this particular instance, help in the battle against tuberculosis.

The articulation of national competencies and the fostering of innovation activities in ways which help pave the way from “workbench to market”, are being supported by recent policies supporting the “health industrial complex” concept [24], [25]: (i) stimulating the private pharmaceutical sector through the Profarma program of Brazil's National Bank for Economic and Social Development, BNDES (http://www.bndes.gov.br/SiteBNDES/bndes/bndes_en/); (ii) supporting the creation of technological development centers in the public sphere, for example, the Center for Technological Development in Health at FIOCRUZ, CDTS [26], [27]; (iii) collaborating with partnership for product development (PDPs) such as FIND and the TB Alliance in the development of diagnostic tools and clinical trials of new drug regimens; (iv) joining open source initiatives addressing drug discovery for neglected tropical diseases [28] as did Fiocruz partnering with WIPO Re:Search (http://www.wipo.int/research/en/). These efforts have been successful in attracting innovative international industries and partners which, although possessing various patent filings in Brazil (Figure 6), are not at present accustomed to developing their R&D&I activities in the country but might in the near future consider Brazil as an option to their international R&D&I strategies. In these contexts partnerships with initiatives of other emerging countries, such as India's open source drug discovery (OSDD) initiative [29], should be promoted. India, aside from sharing the combat of tuberculosis and its drug-resistant forms as a common challenge [9], [30], [31], has been developing inventions in this area and was the third country, after the US and Brazil, in the number of patent applications in Brazil related to the treatment and diagnosis of tuberculosis (Figure 5).

This coverage of joint analyses between publications and patents has shown itself to be particularly important in countries like Brazil that have consolidated their scientific and publication base and, at the moment, count on the same actors to lead technological innovation challenges.

Studies of this nature should be applied to other technological fields to contribute to the betterment of public policy planning and innovation management through the mapping of actors and key institutions and the identification of bottlenecks in the process of generating and granting patent protection to new products and processes.

## Methods

### Co-authorship networks

#### Data mining

Publications by Brazilian authors on tuberculosis were retrieved as raw data files from the ‘Web of Knowledge’ database of the Institute for Scientific Information (ISI), a database that lists the full addresses of all authors of every paper. Queries were made in “advanced search” mode directed simultaneously at the country name and at words in the titles of the papers [e.g. Country  =  Brazil AND Title  =  tuberculosis] to retrieve papers with at least one researcher from Brazil among the authors and having “tuberculosis” in the title.

#### Standardization of names and addresses of authors and institutions

The ISI raw data files were imported into the text-mining software VantagePoint (http://www.thevantagepoint.com) with the appropriate ISI filters. A process of standardization was carried out to bring together the various different names of a particular author or institution and VantagePoint thesauri for names and addresses were created in order to process additional name and address lists.

#### Network assembly, visualization and analysis

Co-occurrence matrices of authorship data were built into VantagePoint and data exported to UCINET software for social network analysis [32]. A co-occurrence matrix shows the number of records in the dataset containing two given list items. Symmetrical, co-occurence matrices (also called “adjacency matrices”) were created using the same set of authorship data in rows and columns in order to map co-authorships between authors (authors×authors matrices) or institutions (institutions×institutions matrices). Networks were assembled, visualized and analyzed for several parameters such as network componentes, cut-points and betweenness with the softwares NetDraw which is embedded in the UCINET package.

### Patent survey

To conduct a survey of all the patent documents for tuberculosis medications and diagnostic kits filed in Brazil, two public databases were consulted: the Brazilian INPI database accessible on the web at http://www.inpi.gov.br and the Espacenet database of the European Patent Office at http://ep.espacenet.com. For the Brazilian base, data collection was done on April, 26, 2010, and it consisted in searching for all documents containing the word tuberculosis or words derived from its root (tuberculo*) in the title or abstract that were applied starting January 1, 1995, which was the date when Brazil began granting patents for pharmaceutical chemicals and medications. The analysis of the evolution of the legal status of patent applications related to Brazilian nationals was done on January 4, 2012. The Espacenet database was searched for documents published in Brazil with the A61P 31/06 classification which according to the IPC pertains to specific therapeutic activity of chemical compounds or medicinal preparations for tuberculosis. This classification was included in the seventh edition of the IPC which entered in vigor on January 1, 2000.

The search of patent information and the comparison of data between the most diverse nations has been especially facilitated as the structure of patent documents and bibliographical data must comply with international standards established by the World Intellectual Property Organization (WIPO). A numerical code, known as the “international agreed numbers for identification of data”(INID) serves as a standardized way to identify all the fields of information present on the cover sheet of the patent document. In addition, the search for technological fields has also been facilitated by the fact that practically all the patent documents around the world are classified according to the International Patent Classification (IPC). The IPC treaty was signed in the city of Strasbourg in 1971 for the purpose of systematically organizing and uniformly classifying patent documents by technological sector of member countries in a manner that facilitates the search and retrieval of this data. Its present hierarchical structure encompasses 8 sections, 129 classes, 639 subclasses and more than 69,100 groups. Beginning with the eighth edition which came into force on June 1, 2006, it underwent substantial changes, better enabling electronic search engines' access to the patent database environment [33].
